# Malaria screening at the workplace in Cameroon

**DOI:** 10.1371/journal.pone.0225219

**Published:** 2019-12-10

**Authors:** Christian Nchetnkou Mbohou, Loick Pradel Kojom Foko, Hervé Nyabeyeu Nyabeyeu, Calvin Tonga, Larissa Kouodjip Nono, Lafortune Kangam, Godlove Wepnje Bunda, Isabelle Matip Mbou, Etoile Odette Ngo Hondt, Alex Joel Koumbo Mbe, Nicolas Policarpe Nolla, Leopold Gustave Lehman

**Affiliations:** 1 Parasitology and Entomology Research Unit, Department of Animal Biology, Faculty of Science, The University of Douala, Douala, Cameroon; 2 Department of Animal Biology, Faculty of Science, University of Yaoundé I, Douala, Cameroon; 3 Department of Zoology and Animal Physiology, Faculty of Science, University of Buea, Buea, Cameroon; 4 Department of Biochemistry, Faculty of Science, The University of Douala, Douala, Cameroon; 5 Faculty of Medicine and Pharmaceutical Sciences, The University of Douala, Douala, Cameroon; Academic Medical Centre, NETHERLANDS

## Abstract

Malaria remains a major health problem in Cameroon; It accounts for 38% of consultations, 24% of deaths and 36.8% of absenteeism in the country. The negative economic impact of malaria has encouraged a new control approach targeting companies. In this regard, a cross sectional study was conducted from February 2015 to June 2017 in 14 companies in the town of Douala. This study aimed at determining the prevalence, control practices of employees and identifying associated factors with malaria. A total of 2705 workers were interviewed and systematically screened for malaria using LED fluorescence microscopy (CyScope®). All positive cases were given a malaria treatment. The prevalence of malaria and asymptomatic malaria was 30.1% and 28.9% respectively; asymptomatic malaria accounted for 95.7% of all positive diagnostic test. Malaria infection was significantly higher in employees aged 36–60 years (30.5%) and having completed primary studies (36%). ITNs ownership and utilization were 86.36% and 77.23% respectively. The risk for malaria infection has significantly decreased with age and educational level while the employees’ level of education and size of households were significantly associated with the regular utilization of ITNs. This is the first study assessing malaria prevalence and risk factors in workplace in Cameroon and using a novel diagnostic tool. This study outlines a high prevalence of malaria infection, especially asymptomatic carriage, high rates of ITNs ownership and utilization, as well as the influence of level of education, age and household size as associated factors. Active case detection of asymptomatic carriers through systematic screening of employees at workplace and their treatment is feasible with the Cyscope microscope and could be a good complement to ongoing control strategies.

## Introduction

Malaria remains the first endemic parasitic disease in the world. The disease is caused by parasites belonging to *Plasmodium* genus which are transmitted to humans through the bite of infected female *Anopheles* mosquitoes. Five *Plasmodium* species are currently involved in malaria cases including *P*. *vivax*, *P*. *malariae*, *P*. *ovale*, *P*. *knowlesi* and especially *P*. *falciparum* the main malarial species [1].

In 2017, malaria was responsible for an estimated 219 million clinical cases of illness and 435,000 deaths worldwide, with the majority of cases (92%) and deaths (93%) reported in sub-Saharan Africa (SSA) [1]. This disease is a hindrance to national growth in several African countries as it is felt in many life areas including education [2,3], agriculture [4], and especially workplace [5]. According to a report issued in 2011 by the Roll Back Malaria (RBM) consortium, malaria directly affects business turnover (increased health care costs) and indirectly the economic environment (increased absenteeism, decreased productivity, weakening of human capital, loss of savings, decrease in investments and tax revenues, reduction of the public health budget) [6].

The situation has improved in recent years through the scaling up of disease control efforts by governments and various stakeholders [7,8,9]. Many malaria control strategies have been implemented [8], as well as other activities such as insecticide-treated mosquito net mass distribution campaigns [10], indoor residual spraying [11] and setting up a parasitology laboratory [12].

Companies have been proposed as important stakeholders to efficiently fight against malaria [6]. They have greatly helped to accelerate the fight against malaria through funding, research and the implementation of many workplace control strategies [6]. In Cameroon, these strategies include the free distribution of long-lasting insecticide-treated nets (LLINs) to employees and their relatives, management of malaria cases and awareness initiatives toward malaria prevention. Recently, our research team has proposed the active detection through new diagnostic tools and treatment of all malaria cases could be an interesting approach. This approach allows for early detection and management of malaria infected individuals especially asymptomatic carriers who greatly fuel the transmission of malaria parasites in endemic areas [2]. We demonstrated previously the utility of fluorescence-based microscopy techniques to achieve this early detection of malaria cases [2]. Fluorescence techniques especially the Cyscope rapid malaria test has increasingly used by Cameroonian authors given their advantages as compared to Giemsa-based techniques [13,14]. The Cyscope microscope is easy-to-use and has dotted with in-built battery allowing for diagnosis of malaria regardless any power supply. In addition, the technique is rapid (5 minutes on average) which allow for mass diagnosis campaigns [2].

To the best of our knowledge no study has addressed the epidemiology of malaria in workplace in Central Africa region using fluorescence microscopy technique. We therefore conducted a study aimed at determining the prevalence and factors associated with malaria infection, determining the prevalence of asymptomatic malaria, determining the practices of employees towards malaria prevention, and identifying factors associated with the utilization of ITNs.

## Materials and methods

### Study area

This cross-sectional study was conducted from February 2015 to June 2017 in 14 companies belonging to 12 activity sectors in the city of Douala (Littoral Region, Cameroon) (Table 1). Located at 4°2’53’N, 9°42’15 E, Douala is the economic capital city and accounts for 35.1% of companies in Cameroon [15]. It consists of six districts namely Douala 1 to 6. Douala is a city where frequent transmission of malaria occurs [16]. This city has a warm and humid climate with temperatures around 26°C and very heavy rainfall, especially during the rainy season that extends from June to October [17]. These climatic conditions are conducive for mosquito development and malaria transmission [18].

**Table 1:** Companies distributed by branch of activity and location in the town of Douala.

Company code	Branch of activity	Location
Com 1	Export cocoa and coffee	Douala 1
Com 2	Security	Douala 1
Com 3	Electricity	Douala 1
Com 4	Food	Douala 3
Com 5	Security	Douala 1
Com 6	Employment agency	Douala 1
Com 7	Security	Douala 1
Com 8	Hydraulic and drilling	Douala 1
Com 9	Public hygiene and sanitation	Douala 3
Com 10	Hotel	Douala 1
Com 11	Construction and public works	Douala 3
Com 12	Manufacturing and selling mattresses and foam	Douala 3
Com 13	Distributing of petroleum products	Douala 1
Com 14	Car Dealership	Douala 3

### Study design

This was a cross-sectional study involving employees of companies based in Douala. Before the study, managers of each company were approached, the aims of the study were explained and dates were proposed for activities in their sites. After obtaining written approval of the company, officials of the communication department in each company informed employees about the study. The participants meeting the eligibility criteria were included in the study upon signing an informed consent form. A pre-tested and structured questionnaire was used to document information of interest of each participant. The presence of malaria parasites was detected using the Cyscope rapid malaria test and malaria-infected persons were treated on the spot.

### Study population and sample size

The study population consisted of employees of each of the abovementioned companies. The following criteria were used to include individuals in this study: i) any employee of the company, ii) of both sexes, iii) aged ≥ 19 years old, iv) having given their assent by signing an informed consent form. Employees who did not fulfill these criteria were not enrolled in the study.

Participants were recruited in a consecutive manner in order to reduce selection bias. Based on a malaria prevalence of 45.47% with fluorescence microscopy as reported by Lehman et al. [2], the minimum sample size was determined using the Lorentz’s formula n = Z^2^pq/d^2^ where p = assumed malaria prevalence among employees, q = 1-p: proportion of malaria negative employees, n = required sample size, Z = statistic for the desired confidence level (1.96 for 95% confidence level) and d = accepted margin of error (5%). The estimated minimum sample size was estimated was n = 381. A total of 2705 employees were included in the study.

### Collection of personal and clinical data

A structured questionnaire was designed to document sociodemographic information and malaria prevention practices among employees respectively (S1 File). The questionnaire was written in English and French, the two national languages in Cameroon. It was primarily pre-tested among 15 employees in order to appraise if it was comprehensible. The inadequate answers were recorded during the pre-test were analyzed and concerned questions were adjusted accordingly and then validated. The final questionnaire was administered to each employee by the principal investigator with the assistance from associate investigators. The interviews lasted between 5–10 minutes and was strictly confidential in order to avoid response bias. After completion of the interview each employee was educated on malaria based on their wrong answers.

### Malaria diagnosis using the CyScope rapid malaria test

CyScope fluorescence microscopy was used for the diagnosis of malaria as described by Lehman et al. [2]. This fluorescence microscope uses readily-prepared and ready to use test slides labeled with a DNA-binding fluorochrome namely 4’,6-Diamidino-2- Phenylindole (DAPI), for detection of intraerythrocytic *Plasmodium* DNA at 443 nm wavelength (UV light) [19, 20]. Briefly, a drop of capillary blood (10 μL) was collected by finger pricking and put on the DAPI-containing area of labeled slides provided by the manufacturer (Partec-Sysmex® GmbH, Germany). The slides were then covered using a cover slip, incubated in dark at room temperature for one minute and then observed under x 40 objective. The presence of bright shiny intracellular tiny dots observed under UV light indicates the presence of malaria parasites in red blood cells [2]. Temperature of each employee was recorded using a digital thermometer before blood collection. Fever was considered as armpit temperature ≥ 37.5°C [2, 21]. Asymptomatic malaria was defined as the presence of malaria parasite with an axillary temperature < 37.5°C while symptomatic malaria was defined as the presence of malaria parasites with an axillary temperature ≥ 37.5°C [2].

### Ethical statements

Ethical clearance was obtained from the University of Douala Institutional Review Board (CEI218 DU/268/05/2019/T). The study was carried out in accordance with guidelines for human experimental models in clinical research as stated by the Cameroon Ministry of Public Health. Written consent was obtained from company managers in the framework of the collaborative with the NGO CCA/SIDA (*Coalition de la Communauté des Affaires contre le SIDA*, *la tuberculose et le paludisme*) that accompanied the study. A code was attributed to each company to guarantee the respect for anonymity of each company. Participation was strictly voluntary, anonymous and without compensation. The objectives of the study were explained to employees in French or English depending on the language they understood best, and their questions were answered. Written informed consent was obtained from all participants prior to enrolment (S2 File). Negative malaria cases with fever were referred to the company’s health center for further examination. All positive cases were treated on the spot with artemisinin based combination therapy (Artesunate-Amodiaquine) as recommended in the national treatment guidelines from the Ministry of Public Health. Written authorizations were obtained from Director/manager of each company. During the study, we also obtained written informed consent from each participant prior to their inclusion in the study. The informed consent forms were written in the two national languages in Cameroon namely English and French.

### Statistical analysis

Data were entered into an Excel spreadsheet, double checked for consistency and analyzed using SPSS version 16.0 software (SPSS Inc., Chicago, IL, USA) (S3 File). Qualitative and quantitative variables were expressed as percentage and mean ± standard deviation (SD) respectively. Pearson’ chi-square (χ^2^) test was used to assess association between variables. Multivariate logistic regression analysis was used to identify factors associated with malaria infection and use of ITNs. Adjusted Odds Ratios (aOR) as well as their 95% Confidence Intervals (CI) were computed. A *P*-value <0.05 was considered as statistically significant.

## Results

### Demographic characteristics of the study population

A total of 2705 employees (458 females and 2247 males) were recruited in the study as shown in Table 2. The mean age of the study population was 37.33 ± 9.78 years old (range: 19–87). The mean temperature at enrolment was 36.37 ± 0.54°C (range: 30–39). Most of participants were aged 36–60 years old (50%), had completed a secondary level of education (52.7%), were workers (80.4%) and were working during the day (56.3%). More than 20% of the participants were living in houses whose household size was more than 7 individuals.

**Table 2:** Demographic characteristics of the participants included in the study.

Variables	COM 1 n = 104	COM 2 n = 78	COM 3 n = 44	COM 4 n = 276	COM 5 n = 275	COM 6 n = 113	COM 7 n = 178	COM 8 n = 132	COM 9 n = 408	COM 10 n = 147	COM 11 n = 104	COM 12 n = 65	COM 13 n = 143	COM 14 n = 638	TOTAL N = 2705
**Gender**															
Female	13(12.5)	14(17.9)	7(15.9)	53(19.2)	39(14.2)	44(38.9)	23(12.9)	12(9.1)	7(1.7)	45(30.6)	10(9.6)	10(15.4)	99(69.2)	137(21.5)	458(16.9)
Male	91(87.5)	64(82.1)	37(84.1)	223(80.8)	236(85	69(61.1)	155(87.1)	120(90.9)	401(98.3)	102(90.4)	94(90.4)	55(84.6)	44(30.8)	501(78.5)	2247(83.1)
**Age (years)**															
[19–36[	57(54.8)	38(48.7)	20(45.5)	138(50.0)	137(49.8)	60(53.1)	92(51.7)	52(39.4)	188(46.1)	72(49.0)	56(53.8)	30(46.2)	54(37.8)	313(49.1)	1307(48.3)
[36–60[	46(44.2)	39(50.0)	24(54.5)	135(48.9)	128(46.5)	51(45.1)	85(47.8)	75(56.8)	220(53.9)	69(46.9)	45(43.3)	34(52.3)	88(61.5)	312(48.9)	1351(50.0)
≥60	01(1.0)	01(1.3)	00(0.0)	3(1.1)	10(3.6)	02(1.8)	1(0.6)	5(3.8)	00(0.0)	6(4.1)	3(2.9)	1(1.5)	01(0.1)	13(2.0)	47(1.7)
**Level of education**															
Primary	13 (12.5)	20 (25.6)	01(2.3)	16(5.8)	86(31.3)	10(8.8)	28(15.7)	25(18.9)	139(34.1)	12(8.2)	17(16.3)	13(20.0)	02(1.4)	74(11.6)	456(16.9)
Secondary	65 (62.5)	48(61.5)	26(59.1)	147(53.3)	158(57.5)	28(24.8)	123(69.1)	67(50.8)	228(55.9)	84(57.1)	53(51.0)	36(55.4)	54(37.8)	308(48.3)	1425(52.7)
University	26 (25.0)	10 (12.8)	10(38.6)	113(40.9)	31(11.3)	75(66.4)	27(15.2)	40(30.3)	41(10.0)	51(34.7)	34(32.7)	16(24.6)	87(60.8)	256(40.1)	824(30.5)
**Districts**															
Douala 1	27(26.2)	22(28.6)	3(7.0)	15(5.5)	52(19.5)	32(28.6)	33(19.1)	12(9.2)	13(3.3)	29(20.0)	8(7.7)	12(18.5)	26(18.6)	41(6.5)	325(12.2)
Douala 2	3(2.9)	10(13.0)	2(4.7)	22(8.1)	72(27.0)	8(7.1)	30(17.3)	6(4.6)	23(5.7)	6(4.1)	3(2.9)	2(2.9)	1(0.7)	33(5.3)	221(8.3)
Douala 3	28(27.2)	28(36.4)	20(46.5)	169(61.9)	80(30.0)	23(20.5)	60(34.7)	74(56.9)	293(72.9)	53(36.6)	75(72.1)	30(46.2)	46(32.9)	366(58.5)	1345(50.6)
Douala 4	12(11.7)	6(7.8)	3(7.0)	8(2.9)	28(10.5)	18(16.1)	21(12.1)	12(9.2)	11(2.7)	12(8.3)	6(5.8)	0(0.0)	13(9.3)	24(3.8)	174(6.5)
Douala 5	33(32.0)	8(10.4)	12(27.9)	58(21.2)	34(12.7)	30(26.8)	24(13.9)	25(19.2)	58(14.4)	43(29.7)	12(11.5)	20(30.8)	51(36.4)	160(25.6)	568(21.4)
Outside of Douala	0(0.0)	3(3.9)	3(7.0)	1(0.4)	1(0.4)	1(0.9)	5(2.9)	1(0.8)	4(1.0)	2(1.3)	0(0.0)	1(1.6)	3(2.1)	2(0.3)	27(1.0)
**Household size**															
≤2	29(28.2)	21(26.9)	12(27.3)	85(30.8)	44(16.8)	20(18.0)	33(18.6)	26(19.7)	76(19.1)	31(21.1)	13(13.1)	12(19.0)	30(21.3)	114(18.3)	546(20.6)
[3–4[	24(23.3)	17(21.8)	11(25.0)	69(25.0)	74(28.2)	36(32.4)	59(33.3)	41(31.1)	110(27.6)	48(32.7)	33(33.3)	17(27.0)	41(29.1)	181(29.1)	761(28.7)
[5–6[	26(25.2)	20(25.6)	10(22.7)	71(25.7)	85(32.4)	34(30.6)	48(27.1)	30(22.7)	122(30.7)	41(27.9)	20(20.2)	19(30.2)	34(24.1)	176(28.3)	735(27.7)
≥7	24(23.3)	20(25.6)	11(25.0)	51(18.5)	59(22.5)	21(18.9)	37(20.9)	35(26.5)	90(22.6)	27(18.4)	33(33.3)	15(23.8)	36(25.5)	152(24.4)	612(23.1)
**Professional category**															
Workers	82(78.8)	74(94.9)	28(63.6)	223(80.8)	260(94.5)	55(48.7)	159(89.3)	94(71.2)	381(93.4)	110(74.8)	70(67.3)	49(75.4)	54(37.8)	537(84.2)	2176(80.4)
Managers	22(21.2)	4(5.1)	16(36.4)	53(19.2)	15(5.5)	58(51.3)	19(10.7)	38(28.8)	27(6.6)	37(25.2)	34(32.7)	16(24.6)	89(62.2)	101(15.)	529(19.6)
**Work time**															
Day	62(59.6)	27(34.6)	25(56.8)	120(43.5)	98(35.6)	79(69.9)	57(32.0)	77(58.3)	188(46.1)	69(46.9)	73(70.2)	49(75.4)	81(56.6)	517(81.0)	1522(56.3)
Night	42(40.4)	51(65.4)	19(43.2)	156(56.5)	177(64.4)	34(30.1)	121(68.0)	55(41.7)	220(53.9)	78(53.1)	31(29.8)	16(24.6)	62(43.4)	121(19.0)	1183(43.7)

Data are presented as frequencies and percentages ().

### Malaria preventive practices

Most of the respondents (84.84%; N = 2705) used at least one malaria preventive method. ITNs was the most frequent method used by the respondents (77.23%) followed by insecticide sprays (17.4%) and cleaning of the environmental (12.7%) as presented in Table 3. Thus, the rate of using ITNs among respondents was 77.23% (2089/2705). The rate of ownership of ITNs was 86.36% (2336/2705), showing that 9.13% of owners of ITNs were not actually using them. The reasons of non-utilization of ITNs included heat (281 citations, 54.24%), work in the night (110 citations, 21.24%), forgetting (53 citations, 10.23%), allergy (42 citations, 8.11%) and damaged ITN (32 citations, 6.18%).

**Table 3:** Malaria prevention methods used by respondents.

Variables	Frequency	Percentage
Methods prevention currently used*		
ITNs	2089	77.23
Insecticide sprays	469	17.40
Long sleeve clothes	409	15.12
Environmental sanitation	345	12.75
Fan/air conditioner	125	4.62
Window/door nets	35	1.30
Repellent body cream	20	0.74
**Total**	**3492**	**100.0**
**Ownership of ITNs**		
Yes	2336	86.36
No	369	13.64
**Total**	**2705**	**100.0**

*: More than one prevention methods can be used by respondents

ITNs: Insecticide-treated bed nets

### Prevalence of malaria and asymptomatic malaria

Two (2) slides were unreadable; parasitological results were thus available for 2703 employees. In total, 815 slides were positive for malaria parasites giving an overall prevalence of 30.1%. The overall prevalence of asymptomatic malaria was 28.9% (780/2703). Asymptomatic cases accounted for 95.71% (780/815) of all positive diagnostic test.

### Malaria prevalence with respect to the company

**Fig 1:**
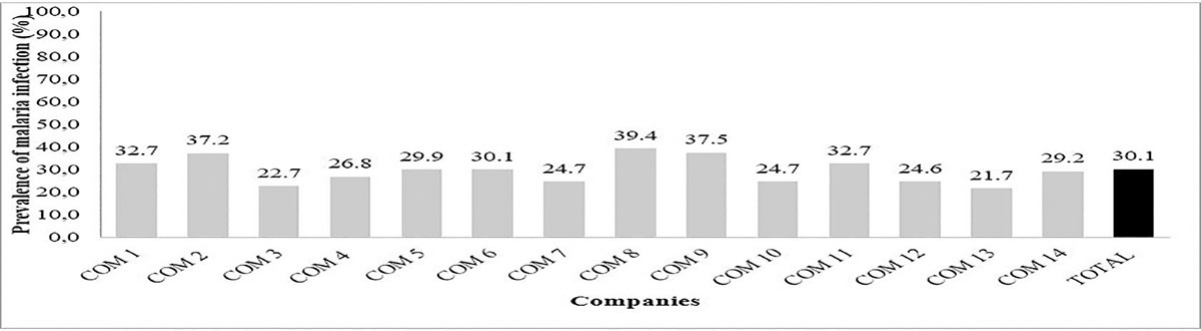
Prevalence of malaria infection with respect to company.

The prevalence of malaria infection varied significantly with regard to the company (χ^2^ = 27.48; df = 11; P = 0.0039). Globally, malaria infection rates were higher than 20% with the highest value reported in Company 8 (39.4%) and lowest value found in Company 13 (21.7%) (Fig 1).

### Factors associated with malaria infection

Three factors namely age, level of education and residence were found to be significantly associated with malaria infection (Table 4). The risk for malaria infection was lower in employees aged ≥60 years old as compared with those aged between 19–36 years old (OR = 0.39; 95%CI: [0.16–0.94]; P = 0.036). Similarly, the risk was lower in employees having completed secondary and university studies (OR = 0.69; 95%CI: [0.53–0.89]; P = 0.004 and OR = 0.72; 95%CI: [0.51–0.99]; P = 0.048 respectively) as compared with those having completed primary studies. In contrast, the risk of infection with malaria parasites was 1.54 times higher in employees from company 9 (working in the Public Hygiene and Sanitation branch) (OR = 1.54; 95%CI: [1.05–2.27]; P = 0.028; Company 4 as Reference group, employees working in the Food branch). No statistically significant association was found between malaria infection and the other factors (gender, professional category, district of residence, fever, and use the various preventive methods assessed).

**Table 4:** Multivariate analysis of factors associated with malaria infection among employees.

Factors	N° of employees examined	N° of employees malaria infected	aOR (95%CI)	P-value
**Gender**				
Female	458	138 (30.1)	1	
Male	2245	677 (30.2)	0.83 (0.64–1.07)	0.154
**Age (years)**				
[19–36[	1306	396 (30.3)	1	
[36–60[	1350	412 (30.5)	0.97 (0.80–1.17)	0.737
≥60	47	7 (14.9)	0.39 (0.16–0.94)	0.036*
**Level of education**				
Primary	456	164 (36.0)	1	
Secondary	1424	407 (28.8)	0.69 (0.53–0.89)	0.004*
University	823	244 (29.6)	0.72 (0.51–0.99)	0.048*
**Professional category**				
Managers	528	160 (30.3)	1	
Workers	2175	655 (30.1)	1.20 (0.89–1.61)	0.225
**Work in the night?**				
No	1182	355 (30.0)	1	
Yes	1521	460 (30.2)	0.88 (0.71–1.11)	0.284
**Branch of activity**				
Food	276	74 (26.8)	1	
Employment agency	113	34 (30.1)	0.89 (0.50–1.60)	0.698
Construction and public works	104	104 (32.7)	1.23 (0.71–2.15)	0.464
Export cocoa and coffee	104	104 (32.7)	1.34 (0.78–2.30)	0.285
Car Dealership	638	186 (22.8)	1.12 (0.78–1.60)	0.538
Security	530	155 (29.2)	1.08 (0.74–1.59)	0.692
Hotel	146	36 (24.7)	0.95 (0.56–1.62)	0.851
Hydraulic and drilling Public	132	52 (39.4)	1.46 (0.88–2.41)	0.143
Hygiene and sanitation	408	153 (37.5)	1.54 (1.05–2.27)	0.028*
Manufacturing and selling mattresses and foam	65	16 (24.6)	0.68 (0.33–1.42)	0.306
Distributing and marketing of petroleum products	143	31 (21.7)	0.76 (0.44–1.30)	0.314
Electricity	44	10 (22.7)	0.61 (0.22–1.71)	0.351
**District**				
Douala 1	325	93 (28.6)	1	
Douala 2	221	72 (32.6)	0.91 (0.59–1.39)	0.649
Douala 3	1343	417 (31.0)	0.97 (0.71–1.31)	0.830
Douala 4	174	54 (31.0)	1.31 (0.84–2.04)	0.239
Douala 5	568	162 (28.5)	0.95 (0.67–1.33)	0.751
Outside of Douala	27	3 (11.1)	0.28 (0.06–1.26)	0.097
**Fever**				
Yes	2087	628 (30.1)	1	
No	616	187 (30.4)	0.94 (0.60–1.45)	0.767
**Use of ITNs**				
Yes	2087	628 (30.1)	1	
No	616	187 (30.4)	1.03 (0.85–1.24)	0.789
**Use of Insecticide sprays**				
Yes	363	105 (28.9)	1	
No	2340	710 (30.3)	1.15 (0.90–1.48)	0.266
**Use of Long sleeve clothes**				
Yes	54	795 (30.0)	1	
No	2649	19 (35.2)	1.10 (0.81–1.48)	0.549
**Environmental Sanitation**				
Yes	345	100 (29.0)	1	
No	2358	715 (30.3)	0.76 (0.56–1.02)	0.069

aOR: Adjusted odds ratio; 95%CI: Confidence interval at 95%

*: statistically significant at P-value <0.05

### Factors associated with use of ITNs

The employees’ level of education and households size were significantly associated with the utilization of ITNs as shown in Table 5. The utilization of ITNs was 1.56 times higher in employees having completed university studies as compared with their counterparts having completed primary studies only (OR = 1.56; 95%CI: [1.16–2.10], P = 0.003). The ITNs utilization rates were gradually higher in respondents living in households with size over 2 individuals as compared with those living in households with one or two individuals. Indeed, the utilization rate was 1.72, 2.15 and 2.53 times higher in employees living in households of 3–4 individuals (OR = 1.72; 95%CI: [1.33–2.23], P < 0.0001), 5–6 individuals (OR = 2.15; 95%CI: [1.66–2.79], P < 10^−4^) and 7 or more individuals (OR = 2.53; 95%CI: [1.94–3.31], P < 0.0001) respectively (Table 5). No statistically significant association was found between ITNs utilization and the other factors (gender, age group, profession, work time, and residence).

**Table 5:** Multivariate analysis of factors associated with the utilization of ITNs.

Factors	Total	Use of ITNs	Adjusted OR (95%CI)	P-value
**Gender**				
Female	458	352 (76.9)	1	
Male	2247	1737 (77.3)	1.09 (0.86–1.39)	0.471
**Age (years)**				
[19–36[	1307	994 (76.1)	1	
[36–60[	1351	1056 (78.2)	1.07 (0.90–1.28)	0.447
≥60	47	39 (83.0)	0.62 (0.32–1.21)	0.159
**Level of education**				
Primary	456	334 (77.3)	1	
Secondary	1425	1112 (78.0)	1.13 (0.89–1.44)	0.302
University	824	643 (78.0)	1.56 (1.16–2.10)	0.003*
**Professional category**				
Managers	529	413 (78.1)	1	
Workers	2176	1676 (77.0)	0.84 (0.65–1.09)	0.181
**Household size**				
≤2	546	397 (72.7)	1	
[3–4]	761	586 (77.0)	1.72 (1.33–2.23)	<0.0001*
[5–6]	736	736 (77.5)	2.15 (1.66–2.79)	<0.0001*
≥7	610	496 (81.3)	2.53 (1.94–3.31)	<0.0001*
**Work in the Night?**				
No	1522	1192 (78.3)	1	
Yes	1183	897 (75.8)	1.19 (0.95–1.46)	0.137
**Residence**				
Douala 1	325	244 (75.1)	1	
Douala 2	221	164 (74.2)	0.70 (0.48–1.04)	0.077
Douala3	1345	1072 (79.7)	0.81 (0.61–1.07)	0.129
Douala4	174	142 (81.6)	0.72 (0.47–1.11)	0.134
Douala 5	568	413 (72.7)	0.99 (0.72–1.35)	0.927
Out of Douala	27	18 (66.7)	0.57 (0.22–1.48)	0.248

Multivariate logistic model was used to compute the adjusted values of odds ratio (aOR). 95%CI: Confidence interval at 95%; ITNs: Insecticide-treated nets;

*: Statistically significant at p-value < 0.05

## Discussion

This is the first study aimed at assessing malaria prevalence and risk factors in workplace in Cameroon. The prevalence of malaria infection was 30.1% in this study with higher prevalence in the hydraulic and drilling company (39.4%) (p = 0.0039). This high prevalence can be justified by the fact that malaria is endemic in Douala [22, 23]. Our result was slightly lower than that obtained by Tchicaya *et al*. (2014) who reported a malaria prevalence of 33% in a study conducted in an electricity company in Ivory Coast [23].

We found a statistically significant association between malaria, age and the level of education of employees. Indeed, the prevalence of malaria decreased with age. This is consistent with findings elsewhere outlining that the risk of malaria decreases with increasing age due to better immunity in older individuals [2, 24, 25, 26, 27]. In highly malaria endemic regions, the risk of repeated malaria episodes is high and individuals living in these areas are exposed to a large variety of malarial antigens that first elicit an immune response against clinical manifestations of malaria and then against parasite growth [24]. As a consequence, clinical malaria (i.e., uncomplicated and severe) are frequent enough in children but rare in adults [28]. This assumption can explain the high rate of asymptomatic carriage of malaria parasites found in the present study. Asymptomatic malaria accounted for 95.71% of all malaria cases and this was consistent with previous reports [2, 13, 29, 30]. Asymptomatic carriage reflects a balance between the host immunity and parasite growth. Asymptomatic carriers represent an important reservoir source for transmission of *Plasmodium* parasites and some studies reported that asymptomatic carriers can greatly fuel malaria transmission [31, 32]. In addition, asymptomatic malaria has been shown to be associated with impaired health status of individuals due to some deleterious effects including chronic anemia, neonatal and maternal mortality, cognitive impairment and increased risk for co-infection with other invasive pathogens such as bacteria [31]. Thus, asymptomatic malaria should be given greater attention in malaria control strategies that might include active case detection of asymptomatic carriers through systematic mass diagnosis campaigns [2]. Active case detection of asymptomatic carrier requests for the utilization of reliable diagnostic tools tailored to field constraints during mass diagnosis campaigns cannot be undermined [2]. In this we used the CyScope fluorescence microscope to study the epidemiology of malaria among employees and that is another peculiarity of our study. This microscope has many advantages over reference methods (i.e., thick and thin blood smears) including diagnostic rapidity (on average 5 minutes to disclose the result), easy-to-use and request little training and expertise [2, 13, 14]. In addition, the CyScope fluorescence microscope is battery operated, making it helpful for field investigation (such active case detection), and difficult-to-reach or remote areas in resource-limited countries where electricity is often lacking [2, 13]. Besides, a study showed the cost-effectiveness and high reliability of this microscope [33].

The level of education of employees appeared to be an important factor to control malaria among workers. We reported significantly lower risk of malaria infection among participants having attended university studies. The level of education has been reported to be a critical determinant for the knowledge of malaria preventive methods as well as participation to malaria control strategies [34, 35]. These facts are in line with reports from this study where the rate and chances for ITNs utilization were both significantly higher in employees with university level as compared to their counterparts with primary level. The risk for malaria infection depends strongly on the exposure of individuals to infectious mosquito bites [36]. ITNs constitute an efficient tool to reduce the chance of contact between humans and infected mosquitoes [37, 38]. ITN, especially LLINs, are one of the cornerstones in the malaria prevention alongside with the management of malaria cases with artemisinin-based combination therapies (ACTs) in most endemic countries [1].

We have found that the prevalence of malaria has significantly varied with regard to companies. The risk of malaria infection was higher in employees from company 9 (Public hygiene and sanitation branch) as compared with those from company 4 (Food branch). This discrepancy is likely due to difference related to management policies of malaria in these two companies. The company 4 implements educational talks on malaria, distributes ITNs to employees and their relatives, and manage laboratory-confirmed febrile malaria cases at their infirmary. In contrast, the policies are lesser developed in the company 9 and consist in the distribution of long-sleeved clothes only. Our finding reinforces the important role of company in the control of malaria.

Besides we did not find any association between gender and malaria infection, this is not consistent with previous reports having outlined the influence of gender in the risk for malaria infection [2, 39–41].

ITN was the main preventive method used by respondents and this can be owed to recent campaign for nationwide mass distribution of LLINs implemented by the Cameroon government since 2011. This finding is consistent with previous reports in the country [36, 42–44]. Over 77% of employees declared use ITNs for malaria prevention. This percentage is lower than 81.8% observed in the overall population of Douala [42]. This difference can be explained by the fact that some employees working at night cannot use ITNs; they use other methods such as long sleeve clothes, insecticidal spray**s** or repellent body cream. Also, this finding on ITNs ownership and utilization rates outlines the importance to promote prevention efforts particularly in relation to changing in attitudes and behavior, training and education action. We reported additional causes of non-utilization of ITNs including damaged ITNs, forgetting and discomfort (heat and allergy). This is consistent with that from previous reports in Cameroon [45] and elsewhere [46, 47]. The non-utilization of ITNs is a particularly challenging situation in malaria control strategies in endemic countries. There is therefore a still important work to do improve the strategies of awareness on benefits of using ITNs and their replacement as damaged in order to prevent malaria among Cameroonian populations.

Interestingly, we showed that ITNs utilization rates were significantly associated with size of households. Employees living in households whose family size was > 2 had more chances of using ITNs compared to their counterparts living in households with family size ≤ 2. This is consistent with a recent report carried out in the Southern part in Cameroon [48]. Sensitization on the benefits of ITNs greatly increases the chances of their usage among owners. Njumkeng and colleagues outlined that the rate of ITNs usage is higher in households with greater number of occupants, as there are more chances to find someone aware on the benefits of ITNs use who can educate their relatives [48]. Conversely, this finding is not in line with previous reports outlining a negative association between family size and ITNs usage [49].

## Conclusion

Findings of this study revealed that malaria remains prevalent at workplace in Douala with a high rate of asymptomatic carriage of malaria parasites. The rates of ITNs ownership was below to that recommended by the WHO with a non-negligible proportion of owners not using their ITNs. The level of education has significantly influenced the prevalence of malaria infection and the rate of ITNs usage among employees. These findings outline the necessity to increase the awareness of employees on the benefits of ITNs. Besides, given the high rate of asymptomatic carriers who constitute a reservoir for malaria transmission, the active detection and drug treatment of these ones using reliable diagnostic tools might reveal an additional interesting control strategies. CyScope fluorescence microscopy could be a valuable tool to achieve this objective.

## Supporting information

S1 File Questionnaire form used to document employees’ data of interest (In English and French). (DOCX)

S2 File Written informed consent form used to include employees (In English and French). (DOCX)

S3 File Database used to make statistical analysis. The name of variables of interest used in the present study were coded (column “Name”) and their labels were specified (column “Label”). COM: Company.
(SAV)

## Abbreviations

ACT: Artemisinin-based combination therapy
CI: Confidence interval
DAPI: 4’,6-Diamidino-2- Phenylindole
Df: Degree of freedom
DNA: Deoxyribonucleic acid
ITNs: Insecticide-treated nets
LED: Light-Emitting Diodes
LLINs: Long-lasting insecticide-treated nets
OR: Odds ratio
RBM: Roll back malaria
SD: Standard deviation
SSA: sub-Saharan Africa
USA: The United States of America
UV: Ultraviolet
WHO: World Health Organization
