# Supplementary material for: Malaria screening at the workplace in Cameroon
Source: PLoS One. 2019 Dec 10;14(12):e0225219. doi: 10.1371/journal.pone.0225219 (PMC6903749; doi:10.1371/journal.pone.0225219)
Supplement: S1 File — (DOCX) [file pone.0225219.s001.docx]

**QUESTIONNAIRE FORM (ENGLISH)**

**Part 1**

Name of company…………………………

ID code ......... District ................................. Age ................ years Gender ......

Level of education ................. Number of people in the household……………………. Occupation ...............

Professional category ……………………………………. Do you work on day or night? …………………………………...

**Part 2**

1. What method of malaria prevention do you practice? ............... ...................................................
2. When you are working in the night, how do you protect yourself from mosquito bites? ..............
3. Regular use of LLIN nets? Yes…………………. No ………………….
4. How many nights have you spent under a mosquito net in the last 3 days? ...................................
   1. Malaria Case Management 1) Hospital 2) Pharmacy 3) Hospital and Pharmacy

**FICHE QUESTIONNAIRE (FRENCH)**

**Partie 1**

Entreprise ………………………

Code ID ………. Quartier …………………………….. Age …………… ans Sexe ………..

Niveau d’étude………………………………………….. Nombre de personnes du ménage…………………

Profession…………………………………………………… Travaillez-vous jour ou la nuit ?.........................

**Partie 2**

1. Quelle méthode de prévention du paludisme pratiquez-vous ?......................................................
2. Comment vous protégez-vous contre les piqûres de moustiques lorsque vous travaillez ? ……………………………………………………………………………………………………………………………………………………..
3. Dormez-vous toujours sous une moustiquaire imprégnée d’insecticide ? …………………………………….
4. Combien de nuit avez-vous passées sous une moustiquaire ces 3 derniers jours ? ……………………… a. Gestion des cas de paludisme 1) Hôpital 2) Pharmacie 3) Hôpital et pharmacie
