# Supplementary material for: Malaria screening at the workplace in Cameroon
Source: PLoS One. 2019 Dec 10;14(12):e0225219. doi: 10.1371/journal.pone.0225219 (PMC6903749; doi:10.1371/journal.pone.0225219)
Supplement: S2 File — (DOCX) [file pone.0225219.s002.docx]

**INFORMED CONSENT:**

Theme of the study: **Malaria screening in the workplace in Cameroon**

certify that I have read (or that someone has read for me) the notice explaining the purpose of the study project and the expectations towards me. The opportunity was offered to me to ask all my questions and satisfactory answers were provided to me. I received a copy of this document. I understand what is expected of me if I agree to participate in the study. I also know that I can suspend my participation in this study at any time without any risk or prejudice. It has also been explained to me that all the personal data that I will provide will be kept in the confidentiality and the respect of the medical secret. BY SIGNING THIS DOCUMENT, I AGREE TO FULL GRE PARTICIPATE IN THE STUDY IT PRESENTED.

**Name of Participant_**_________________________________________ ____________

Signature of the Participant Date

INVESTIGATOR

I explained the study and answered all the questions of the participant. I believe that she understood the information in the notice and that she freely agrees to participate in this study.

________________________________________

**Name of the Investigator**

________________________________________ ______________________________

Signature of the Investigator Date (the same as that of the participant)

**CONSENTEMENT ECLAIRE :**

Thème de l'étude: **Dépistage de masse du paludisme en milieu professionnel au Cameroun**

Certifie que j'ai lu (ou que quelqu'un a lu pour moi) la description du sujet de l'étude et les attentes à mon égard. L'occasion m'a été offerte de poser mes questions et réponses. J'ai reçu une copie de ce document. Je comprends ce que l'on attend de moi si j'accepte de participer à l'étude. Je sais également que je peux suspendre ma participation à cette étude à tout moment sans risque ni préjudice. On m'a expliqué qu'il sera gardé dans la confidentialité et le respect du secret médical. En signant ce document, j'accepte de participer pleinement à l'étude présentée.

**Nom du participant**__________________________________________ ____________

Signature du participant Date

**INVESTIGATEUR**

J'ai expliqué l'étude et répondu à toutes les questions du participant. Je crois qu'elle a compris l'information contenue dans cette brochure et a accepté de participer à cette étude.

________________________________________

Nom de L’INVESTIGATEUR

________________________________________ ______________________________

Signature de L’INVESTIGATEUR Date
